# Inhibitory Effects of Osthole on Human Breast Cancer Cell Progression via Induction of Cell Cycle Arrest, Mitochondrial Dysfunction, and ER Stress

**DOI:** 10.3390/nu11112777

**Published:** 2019-11-15

**Authors:** Wonhyoung Park, Sunwoo Park, Gwonhwa Song, Whasun Lim

**Affiliations:** 1 Department of Biotechnology, Korea University, Seoul 02841, Korea; pwh9224@korea.ac.kr (W.P.); sunwoojump@korea.ac.kr (S.P.); 2 Department of Food and Nutrition, Kookmin University, Seoul 02707, Korea

**Keywords:** breast cancer, osthole, apoptosis, MMP depolarization, calcium imbalance, ER stress

## Abstract

Background: Breast cancer is the most commonly diagnosed cancer and the second leading cause of cancer death in women. Although, recently, the number of pathological studies of breast cancer have increased, it is necessary to identify a novel compound that targets multiple signaling pathways involved in breast cancer. Methods: The effects of osthole on cell viability, apoptosis, mitochondria-mediated apoptosis, production of reactive oxygen species (ROS), and endoplasmic reticulum (ER) stress proteins of BT-474 and MCF-7 breast cancer cell lines were investigated. Signal transduction pathways in both cells in response to osthole were determined by western blot analyses. Results: Here, we demonstrated that osthole inhibited cellular proliferation and induced cell cycle arrest through modulation of cell cycle regulatory genes in BT-474 and MCF-7 cells. Additionally, osthole induced loss of mitochondrial membrane potential (MMP), intracellular calcium imbalance, and ER stress. Moreover, osthole induced apoptosis by activating the pro-apoptotic protein, Bax, in both cell lines. Osthole regulated phosphorylation of signaling proteins such as Akt and ERK1/2 in human breast cancer cells. Furthermore, osthole-induced activation of JNK protein-mediated apoptosis in both cell lines. Conclusions: Collectively, the results of the present study indicated that osthole may ameliorate breast cancer and can be a promising therapeutic agent for treatment of breast cancer.

## 1. Introduction

Breast cancer is the most common cancer-related cause of mortality in United States [1]. The incidence of breast cancer has risen steadily and is expected to continue to rise [2]. Changing reproductive patterns-pregnancy at later ages and decreased duration of breast feeding promote the acceleration of breast cancer pathogenesis [3]. Breast cancer is frequently diagnosed in advanced stages because few symptoms manifest during the early stages of the disease. Diagnosis and treatment strategies have improved, but numerous barriers to treatment—metastasis, drug side effects, and drug resistance—remain significant challenges [4]. Because of the hypoxic tumor environment, approximately half of patients with advanced breast cancer show resistance to radiotherapy [5]. Additionally, heterogeneity of breast cancer presents a significant obstacle to treatment with specific chemotherapeutic agents [6]. Breast cancer cells could be classified into five subtypes based on gene expression profiles: Luminal A, luminal B, HER2-overexpression, basal, and normal-like. Each subtype shows different clinical outcomes [7]. Particularly, luminal A (MCF-7) and luminal B (BT-474) types used in this study are ER positive cell lines which is are usually hormone responsive. However, BT-474 is a HER2 positive and MCF-7 is a HER2 negative cell line [8]. Moreover, luminal A type has been known to have good prognosis because it is highly differentiated whereas luminal B type has a highly proliferative status and shows poorer prognosis than A type [9]. Therefore, a therapeutic strategy that target these multiple cell types is required.

Phytochemicals exhibit low cytotoxicity to normal cells, but may target heterogenous cancer cells and multiple signaling pathways [10]. Studies have shown that phytochemicals exert promising therapeutic effects against breast cancer; e.g., curcumin, resveratrol, and sulforaphane-induced apoptosis and cell cycle arrest and decreased proliferation of breast cancer cells [11,12,13]. Furthermore, studies showed that phytochemicals can target chemo-resistant breast cancer stem cells [14]. Therefore, phytochemicals are considered promising agents for complete treatment of breast cancer. 

The biological effects of coumarin and its derivatives have been extensively studied. Studies have shown that the chemical structure of coumarin contributes to its pharmacological properties [15]. Coumarin derivatives have been used as anticoagulants, anti-HIV agents, and for treatment of CNS diseases [16,17,18]. Osthole (7-methoxy-8-(3-methyl-2-butenyl) coumarin) is a phytochemical from *Cnidium monnieri* (L.) Cusson, which is widely used as a traditional herbal medicine. Osthole is known to exert anti-inflammatory, anti-microbial, and anti-allergic activities [19,20] and has attracted increased attention because of its anti-cancer activity. Osthole is also known to exert therapeutic effects against several cancer types including lung, hepatic, cervical, and ovarian cancer. In addition, osthole induced apoptosis of immortalized hepatocellular carcinoma cells and suppressed hepatic tumor mass growth in mice [21]. Furthermore, osthole inhibited cell proliferation and induced cell cycle arrest in lung and ovarian cancer [22,23]. It exerts anti-cancer effects against breast cancer by attenuating cell proliferation and metastasis [24]. A recent study revealed that osthole suppressed the triple negative breast cancer cell lines by blocking STAT3 signaling pathway [25]. This result supports osthole as having a potential for the management of breast cancer by targeting intracellular signaling pathways. However, the molecular mechanisms of the anticancer effects of osthole in the luminal type of breast cancer cell lines have not been elucidated. 

We aimed to examine the anti-cancer mechanisms of osthole in MCF-7 and BT-474 breast cancer cell lines. We evaluated its anti-proliferative apoptotic effects and investigated the disruption of intracellular calcium levels, mitochondrial membrane potential, and ER stress as well as its effects on signaling molecules in the MAPK and PI3K/Akt signaling pathways. 

## 2. Materials and Methods

### 2.1. Compounds

Osthole (catalog number: O9265) was purchased from Sigma (St. Louis, MO, USA). Osthole was dissolved in DMSO to prepare a chemical stock for treatment. Antibodies against phosphorylated Akt (Ser^473^, catalog number: 4060), P70S6K (Thr^421^/Ser^424^, catalog number: 9204), S6 (Ser^235^/Ser^236^, catalog number: 2211), ERK1/2 (Thr^202^/Tyr^204^, catalog number: 9101), p90RSK (Thr^573^, catalog number: 9346), JNK (Thr^183^/Tyr^185^, catalog number: 4668), total Akt (catalog number: 9272), P70S6K (catalog number: 9202), S6 (catalog number: 2217), ERK1/2 (catalog number: 4695), p90RSK (catalog number: 9335), JNK (catalog number: 9252), IRE1α (catalog number: 3294), eIF2α (catalog number: 5324), Bak (catalog number: 12105S), and Bax (catalog number: 2772) were purchased from Cell Signaling Technology (Beverly, MA, USA). Bcl-xL, p-Bcl-2, cleaved caspase 3 and cleaved caspase 9 were also purchased from cell Signaling Technology. Antibodies against GRP78 (catalog number: sc-13968), ATF6 α (catalog number: sc-166659), and α-tubulin (TUBA, catalog number: sc-32293) were purchased from Santa Cruz Biotechnology, Inc (Santa Cruz, CA, USA). Inhibitors of ERK1/2 (U0126, catalog number: E1282) and JNK (SP600125, catalog number: E1305) were purchased from Enzo Life Sciences, Inc (Farmingdale, NY, USA), and a PI3K/Akt inhibitor (LY294002, catalog number: 9901) was purchased from Cell Signaling Technology, Inc.

### 2.2. Cell Culture

BT-474 and MCF-7 cells (breast cancer cells) were purchased from the Korean Cell Line Bank (KCLB; Seoul, Korea) and cultured in RPMI 1640 with HEPES (catalog number: SH30255.01, HyClone, Logan, UT, USA) containing 10% fetal bovine serum. All cells were incubated at 37 °C in a 5% CO_2_ atmosphere. For use in experiments, monolayers of BT-474 and MCF-7 cells were grown in culture medium to 70–80% confluence in 100-mm culture dishes. The cells were treated with different doses of osthole with or without cell signaling pathway inhibitors. 

### 2.3. Proliferation Assay

Proliferation assays were conducted using a Cell Proliferation ELISA, BrdU kit (catalog number: 11647229001, Roche, Basel, Switzerland) according to the manufacturer’s instructions. Briefly, BT-474 and MCF-7 cells (1 × 10^5^ cells per 100 μL) were seeded in 96-well plates, then treated with osthole (0, 5, 10, 20, 50, and 100 μM). After incubating for 48 h, 10 μM bromo-2′-deoxyuridine (BrdU) was added to each well, and the cells were incubated for 2 h at 37 °C. After labeling with BrdU, the cells were fixed and incubated with anti-BrdU-peroxidase (POD) working solution for 90 min. The anti-BrdU-POD bound to BrdU incorporated into newly synthesized cellular DNA, and these immune complexes were detected following reaction with the 3,3′,5,5′-tetramethylbenzidine (TMB) substrate. Absorbance of the reaction product was determined at 370 and 492 nm using an ELISA reader. The DMSO-treated group was used as negative control.

### 2.4. Cell Cycle Analysis

Cells (1 × 10^5^ cells per 2 mL) were seeded in 6-well plates, then incubated for 24 h in serum-free RPMI 1640 with HEPES. The cells were then treated with different doses of osthole (0, 10, 20, and 50 μM) for 48 h. After treatment, the cells were collected, washed twice with cold 0.1% bovine serum albumin (BSA) in PBS, and fixed with 70% ethanol at 4 °C for 24 h. BT-474 and MCF-7 cells were centrifuged and the supernatant was discarded. Pellets were washed twice with 0.1% BSA-PBS and stained with propidium iodide (PI; BD Biosciences, Franklin Lakes, NJ, USA) and 100 µg/mL RNase A (Sigma–Aldrich, St. Louis, MO, USA) for 30 min in the dark. Fluorescence intensity was analyzed using a flow cytometer (BD Biosciences). The DMSO-treated group was used as negative control.

### 2.5. Immunofluorescence Microscopy

The effects of osthole on the expression of proliferating cell nuclear antigen (PCNA) were determined using immunofluorescence microscopy. Cells (3 × 10^4^ cells per 400 μL) seeded in confocal dishes were incubated with or without osthole (50 μM) for 24 h at 37 °C in a CO_2_ incubator. After treatment with osthole, the cells were fixed with methanol for 10 min at −20 °C and then probed with mouse monoclonal PCNA (catalog number: sc-56, Santa Cruz Biotechnology) at a final dilution of 1:100 (2 μg/mL). The cells were then incubated with goat anti-mouse IgG Alexa 488 (catalog number: A-11001, Invitrogen, Carlsbad, CA, USA) at a 1:200 dilution for 1 h at room temperature. The cells were then washed using 0.1% bovine serum albumin in phosphate-buffered saline (PBS) and overlaid with 4′,6-diamidino-2-phenylindole (DAPI). Images were captured using a LSM710 (Carl Zeiss, Oberkochen, Germany) confocal microscope. The DMSO-treated group was used as negative control.

### 2.6. Annexin V and PI Staining

Osthole-induced apoptosis of breast cancer cells was analyzed using a FITC Annexin V apoptosis detection kit I (BD Biosciences, Franklin Lakes, NJ, USA). The cells (1 × 10^5^ cells per 2 mL) were seeded in 6-well plates and maintained in serum-free medium for 24 h. The cells were then treated with osthole at different doses (0, 10, 20, and 50 μM) for 48 h at 37 °C in a CO_2_ incubator. The cells were collected by centrifugation, washed with PBS, and resuspended using 1× Annexin V binding buffer at 1 × 10^6^ cells/mL. Next, 100 μL of the cell suspensions (1 × 10^6^ cells) was transferred to 1.5-mL culture tubes and incubated with 5 μL FITC Annexin V and 5 μL propidium iodide (PI) for 15 min at room temperature in the dark. Four hundred microliters of 1× Annexin V binding buffer was added to the 1.5 mL culture tubes. Fluorescence intensity was determined using a FACS instrument (BD Accuri C6 Plus). The DMSO-treated group was used as negative control.

### 2.7. TUNEL Assay

BT-474 and MCF-7 cells (3 × 10^4^ cells per 400 μL) were seeded onto confocal dishes (catalog number:100350, SPL Life Science, Gyeonngi-do, Korea), then treated with osthole at a final concentration of 50 μM for 48 h at 37 °C in a CO_2_ incubator. After incubation, the cells were air-dried and fixed with 4% paraformaldehyde in PBS for 1 h at room temperature. The cells were rinsed with PBS briefly and permeabilized with 0.1% Triton X-100 in 0.1% sodium citrate for 5 min on ice. Next, the cells were subjected to terminal deoxynucleotidyl transferase dUTP nick-end labeling (TUNEL) staining using the In-Situ Cell Death Detection Kit, TMR red for 1 h at 37 °C in the dark. The cells were then washed with PBS and counterstained with DAPI (catalog number: D8417, Sigma-Aldrich). Fluorescence was detected using an LSM710 (Carl Zeiss) confocal microscope fitted with a digital microscope AxioCam camera with Zen2009 software. DMSO-treated cells were used as negative control.

### 2.8. JC-1 Mitochondrial Membrane Potential Assay

Changes in MMP were analyzed using a mitochondrial staining kit (catalog number: CS0390, Sigma-Aldrich). Breast cancer cells (1 × 10^5^ cells per 2 mL) were seeded in 6-well plates and treated with osthole at different doses (0, 10, 20, and 50 μM) for 48 h at 37 °C in a CO_2_ incubator. The cells were collected by centrifugation, resuspended in staining solution including 200 × JC-1 and 1 × staining buffer, and incubated at 37 °C in a CO_2_ incubator for 20 min. The stained cells were collected by centrifugation and washed once with 1× JC-1 staining buffer. After washing, the cell suspension was centrifuged, and then the cells were resuspended in 500 µL of staining buffer. Fluorescence intensity was analyzed using a FACS instrument (BD Accuri C6 Plus). The DMSO-treated group was used as negative control.

### 2.9. Measurement of Cytosolic Calcium Influx 

BT-474 and MCF-7 cells (1 × 10^5^ cells per 2 mL) were seeded onto 6-well plates and incubated for 24 h in serum-free medium until the cells reached 70–80% confluence. The cells were treated with different doses of osthole (0, 10, 20, and 50 μM) for 48 h at 37 °C in a CO_2_ incubator. The cells were collected by centrifugation. The collected cells were stained with 3 μM fluo-4 AM (Invitrogen) and incubated at 37 °C in a CO_2_ incubator for 20 min. The stained cells were washed with PBS, and fluorescence intensity was analyzed using a flow cytometer (BD Biosciences). The DMSO-treated group was used as negative control.

### 2.10. Quantitative RT-PCR Analysis

All primers were synthesized by Bioneer (Daejeon, Korea). Each primer was designed using sequences in the GenBank database as illustrated in Table 1. Gene expression levels were measured using a StepOnePlus Real-Time PCR System (Applied Biosystems, Waltham, MA, USA). Sequence-specific products were identified by generating a melting curve. Relative gene expression levels were quantified using the 2^−ΔΔCT^ method. RNA expression in each reaction was normalized to *glyceraldehyde-3-phosphate dehydrogenase* (*GAPDH*) gene expression. The DMSO-treated samples were used as negative control.

### 2.11. Western Blot Analyses

Total protein concentration in whole-cell extracts was determined using Bradford reagent (Bio-Rad, Hercules, CA, USA) using BSA as the standard. Proteins were denatured, separated using sodium dodecyl sulfate-polyacrylamide gel electrophoresis (SDS–PAGE), and transferred to nitrocellulose membranes. Blots were developed using enhanced chemiluminescence detection solutions (Super Signal West Pico, Pierce, Rockford, IL, USA) and quantified by measuring the intensity of light emitted from correctly sized bands under UV light using a Chemi Doc EQ system and Quantity One software (Bio-Rad). Immunoreactive proteins were detected using specific antibodies against phosphorylated and total proteins at a 1:1000 dilution. Total protein or α-tubulin (TUBA) was used to normalize the levels of target proteins. The signal intensity in each dose was represented as the relative value compared to signal intensity in 0 uM.

### 2.12. Statistical Analysis 

All quantitative data was subjected to least squares analysis of variance (ANOVA) using the General Linear Model procedure of Statistical Analysis System software (SAS Institute, Inc., Cary, NC, USA). All significance tests were performed using the appropriate error terms according to the expectation of the mean squares for error. A *p*-value less than or equal to 0.05 was considered statistically significant. Data are presented as least-square means (LSMs) with standard errors (SEs).

## 3. Results

### 3.1. Osthole Selectively Inhibited Cellular Proliferation of Human Breast Cancer Cells

**Figure 1:**
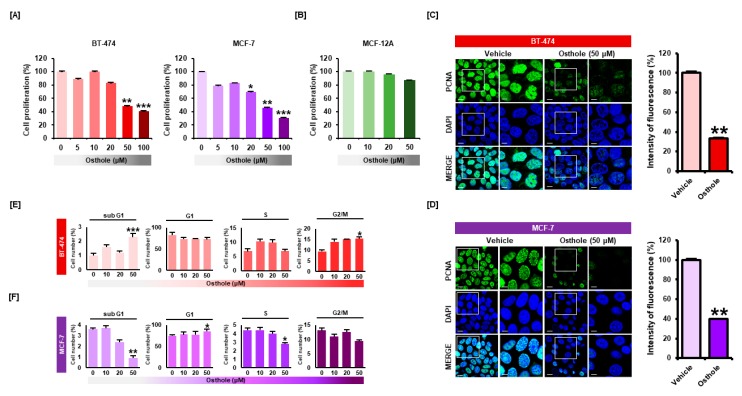
Osthole inhibited cell proliferation in human breast cancer cells. (**A**) Proliferation of breast cancer cells was analyzed using cell proliferation assays. Osthole (0, 5, 10, 20, 50, and 100 μM) decreased the proliferation of BT-474 and MCF-7 cells in a dose-dependent manner. (**B**) Effect of osthole on MCF-12A normal mammary epithelial cell proliferation in a dose dependent manner (0, 10, 20, and 50 μM). (**C**,**D**) Proliferative cell nuclear antigen (PCNA) was detected using immunofluorescence analysis. The green signal associated with PCNA was attenuated in osthole-treated cells compared to vehicle-treated cells. Nuclei were counterstained with DAPI (blue). The graph shows the intensity of PCNA fluorescence. (**E**,**F**) Regulatory effect of osthole on cell cycle progression in breast cancer cells. The number of cells in each cell cycle phase was analyzed by propidium iodide (PI) staining of DNA contents using flow cytometry. All experiments were performed in biological triplicate and the asterisks represent statistically significant differences compared to control (DMSO-treated cells; 0 μM) (* *p* < 0.05, ** *p* < 0.01 and *** *p* < 0.001).

To investigate the effect of osthole on breast cancer cells, a proliferation assay was performed with BT-474 and MCF-7 cells. Osthole reduced cell proliferation in a dose-dependent manner (Figure 1A). Osthole decreased proliferation of BT-474 cells by 51% (*p* < 0.01) at 50 μM and 59% (*p* < 0.001) at 100 μM. Proliferation of MCF-7 cells was decreased by 54% (*p* < 0.01) following 50 μM osthole treatment and 70% (*p* < 0.001) following 100 μM osthole treatment. However, osthole did not affect the cell viability of MCF-12A normal breast epithelial cells (Figure 1B). Based on the results of the proliferation assay, all subsequent experiments were performed using 50 μM osthole. Next, we evaluated the expression of a proliferative marker in breast cancer cells following osthole treatment (Figure 1C,D). Green fluorescent signal associated with PCNA was reduced following osthole treatment in BT-474 (33%) and MCF-7 (39%) cells. To examine the effects of osthole on the cell cycle progression in breast cancer cells, we analyzed cell cycle distribution by monitoring DNA content of BT-474 and MCF-7 cells. The percentages of cells occupying each cell cycle phase were calculated compared with DMSO treated cells. Osthole increased the number of cells at sub-G1 phase 2.3-fold in BT-474 cells (Figure 1E). In MCF-7 cells, osthole increased the number of G1 phase cells and reduced the number of sub-G1 and S phase cells (Figure 1F).

### 3.2. Osthole Downregulated Gene Expression Associated with Cell Cycle Progression in Breast Cancer Cells

**Figure 2:**
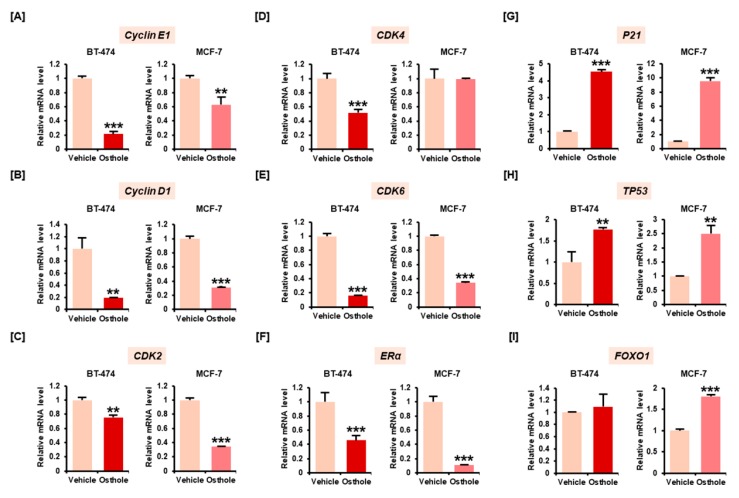
Relative mRNA expression of cell cycle-related genes including *cyclin E1* (**A**), *cyclin D1* (**B**), *CDK2* (**C**), *CDK4* (**D**), *CDK6* (**E**), *ERα* (**F**), *P21* (**G**), *TP53* (**H**), and *FOXO1* (**I**). Expression of cell cycle-related genes in breast cancer cell lines was analyzed by quantitative RT-PCR using cDNA templates based on RNA isolated from BT-474 and MCF-7 cells. The graphs indicate the relative mRNA expression in breast cancer cell lines treated with vehicle and osthole for 24 h. All experiments were performed in technical triplicate and the asterisks represent statistically significant differences compared to vehicle-treated cells (** *p* < 0.01 and *** *p* < 0.001).

We investigated the mRNA expression of cell cycle-related genes—*Cyclin*, *CDK*, *forkhead box protein O1 (FOXO1)*, *P21*, *TP53*, and *ERα*. The expression of *Cyclin D1* (Figure 2A), *Cyclin E1* (Figure 2B), *CDK2* (Figure 2C), and *CDK6* (Figure 2E) were significantly decreased following treatment with 50 μM osthole in BT-474 and MCF-7 cells. The mRNA expression of *CDK4* was decreased only in BT-474 cells (Figure 2D). *Estrogen receptor α* (*ERα*), a key effector in breast cancer progression, was decreased approximately 0.5-fold (*p* < 0.001) and 0.2-fold (*p* < 0.001) in BT-474 and MCF-7 cells, respectively (Figure 2F). The CDK inhibitor *CDKN1A* (also known as *P21)*, and transcription factors associated with cell cycle arrest including *TP53* and *FOXO1*, were elevated following osthole treatment in MCF-7 cells (Figure 2G–I). Similarly, osthole increased the expression of *P21* and *TP53* in BT-474 cells without affecting *FOXO1* expression. These findings suggested that osthole induced cell cycle arrest by modulating gene expression of cell cycle regulators in two different cell types of breast cancer cell lines. 

### 3.3. Osthole Disrupted the Mitochondrial Membrane Potential and Intracellular Calcium Flux in Human Breast Cancer Cells

**Figure 3:**
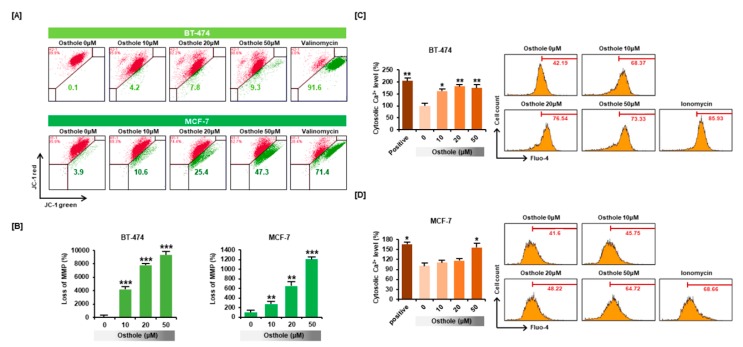
Effects of osthole on mitochondrial dysfunction and cytosolic calcium influx in human breast cancer cells. (**A**,**B**) Mitochondrial membrane potential (MMP) was evaluated in JC-1 stained BT-474 and MCF-7 cells using flow cytometry. Depolarization of mitochondria was indicated by increased green fluorescence in the dot plot. The cells treated with Valinomycin (1 μg/mL) for 20 min were used as a positive control for mitochondrial depolarization. (**C**,**D**) Calcium levels in the cytoplasm were evaluated using the cytosolic calcium indicator, Fluo-4. The cells treated with Ionomycin (12 μM) for 5 min were used as a positive control for calcium flux. All experiments were performed in biological triplicate and the asterisks represent statistically significant differences compared to vehicle-treated cells (* *p* < 0.05, ** *p* < 0.01 and *** *p* < 0.001).

To determine the effects of osthole on intracellular homeostasis in breast cancer cells, we investigated changes in mitochondrial membrane potential (MMP) using the JC-1 assay. The green signal associated with JC-1 monomer was increased in a dose-dependent manner to 9300% (*p* < 0.001) in BT-474 cells and 1212% (*p* < 0.001) in MCF-7 cells, which showed that osthole induced depolarization of the mitochondria membrane in breast cancer cells (Figure 3A,B). Furthermore, osthole gradually increased cytosolic calcium influx in both cell lines (Figure 3C,D). Cytosolic calcium levels increased to 174% (*p* < 0.01) in BT-474 cells and 156% (*p* < 0.05) in MCF-7 cells in response to 50 μM osthole treatment.

### 3.4. Osthole Activated ER Stress Pathways in Human Breast Cancer Cells

**Figure 4:**
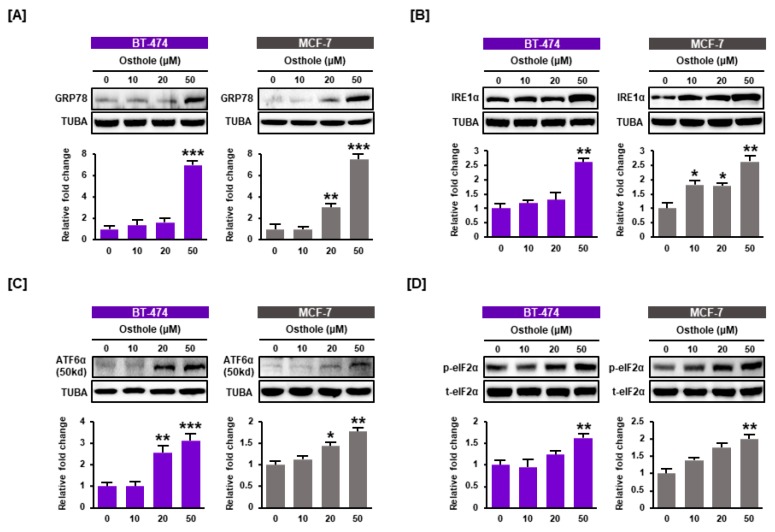
ER stress induced by osthole treatment in human breast cancer cells. The levels of the ER regulatory proteins GRP78 (**A**), IRE1α (**B**), ATF6α (**C**), and eIF2α (**D**) were analyzed using western blot analysis of BT-474 and MCF-7 cells. The levels of GRP78, IRE1α, and ATF6α were normalized to α-tubulin (TUBA), and phosphor eIF2α was normalized to total eIF2α protein. All experiments were performed in biological triplicate and the asterisks represent statistically significant differences (* *p* < 0.05, ** *p* < 0.01 and *** *p* < 0.001).

Due to the finding that osthole disrupted intracellular calcium homeostasis in breast cancer cells, we measured the effect of osthole on the endoplasmic reticulum (ER) using western blot analysis. Osthole upregulated the expression of ER stress-inducing proteins in a dose-dependent manner in BT-474 and MCF-7 cells. The expression of GRP78 was increased by approximately 7-fold (*p* < 0.001) in both cell lines (Figure 4A). Additionally, the expression of IRE1α, cleaved ATF6α, and phospho-eIF2α increased by 2.5-fold, 3-fold, and 1.7-fold, respectively, following treatment with 50 μM osthole (Figure 4B–D). These results showed that osthole induced ER stress in human breast cancer cells. 

### 3.5. Osthole Induced Apoptosis in Human Breast Cancer Cells

**Figure 5:**
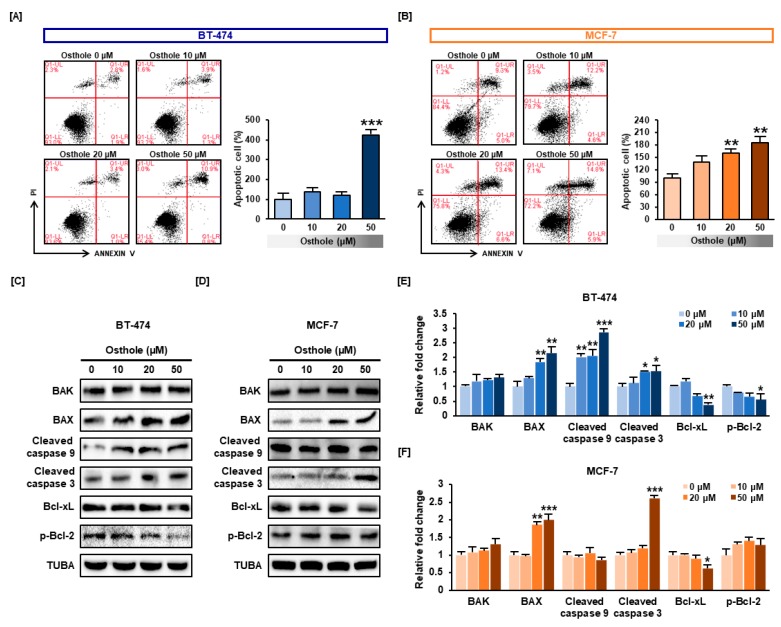
The apoptotic effects of osthole on human breast cancer cells. (**A**,**B**) Apoptotic cells were detected by flow cytometry using Annexin V/PI staining. The graph represents increased numbers of cells in the late apoptotic phase (upper right side of dot plot) in response to osthole (0, 10, 20, and 50 μM). Data were analyzed relative to the DMSO control (0 μM). (**C**,**D**) The expressions of the pro-apoptotic proteins including Bax, Bak, cleaved caspase 3 and cleaved caspase 9, and anti-apoptotic proteins including Bcl-xL and p-BcL-2 were analyzed by western blot in BT-474 and MCF-7 cells. The expression levels of apoptotic proteins in BT-474 (**E**) and MCF-7 (**F**) were represented as bar graphs. The expression levels of proteins were normalized to α-tubulin (TUBA). All experiments were performed in biological triplicate and the asterisks indicate statistically significant differences compared to control (DMSO-treated cells; 0 μM) (* *p* < 0.05, ** *p* < 0.01 and *** *p* < 0.001).

We examined whether osthole induced apoptosis in human breast cancer cells using the Annexin V assay. The number of apoptotic cells increased in an osthole dose-dependent manner to 423% (*p* < 0.001) in BT-474 cells and 186% (*p* < 0.01) in MCF-7 cells (Figure 5A,B). Furthermore, the expression of pro-apoptotic proteins was analyzed using western blot analysis. Osthole treatment resulted in increased the expression of Bax about 2-fold in both cell lines (*p* < 0.001). Contrastingly, Bak was only slightly increased in response to osthole treatment in both breast cancer cells (Figure 5C,D). Moreover, cleaved form of caspase 3 was also increased about 2.6-fold and 1.5-fold in BT-474 and MCF-7 cells. Cleaved caspase 9 was increased by 2.8-fold in MCF-7 whereas not significantly changed in BT-474 cells. Additionally, we investigated the expression of Bcl-xL and phosphorylated Bcl-2 known as antiapoptotic factors. Osthole decreased the expression of Bcl-xL less than 0.63-fold and 0.38-fold in BT-474 and MCF-7 cells. Phosphorylated Bcl-2 was decreased less than 0.56-fold in MCF-7 cells while p-Bcl-2 in BT-474 has not been changed. 

### 3.6. Osthole Regulated the PI3K/Akt and MAPK Signaling Pathways in Human Breast Cancer Cells

**Figure 6:**
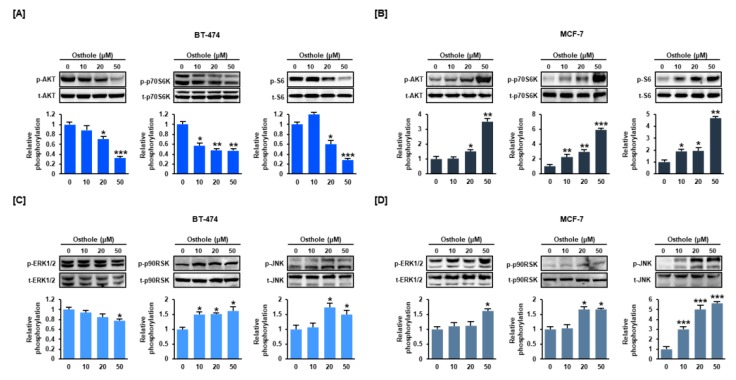
Effect of osthole on phosphorylation of PI3K/Akt and MAPK pathway proteins in human breast cancer cells. The levels of Akt, p70S6K, S6 (**A**,**B**), ERK1/2, p90RSK, and JNK (**C**,**D**) were detected by western blot analysis. Each phosphorylated protein was normalized to the corresponding total protein. Band intensity is shown in the graph as relative value compared to non-treated control (0 μM). All experiments were performed in biological triplicate and the asterisks indicate statistically significant differences (* *p* < 0.05, ** *p* < 0.01 and *** *p* < 0.001).

To determine osthole-mediated cell signaling mechanisms, phosphorylation of signaling proteins associated with the PI3K/Akt and MAPK pathways was detected by western blotting. Osthole inhibited the phosphorylation of Akt, p70S6K, and S6 in BT-474 cells (Figure 6A), but upregulated phosphorylation of these proteins in MCF-7 cells (Figure 6B). Similarly, phosphorylated ERK1/2 was decreased in BT-474 cells, but increased in MCF-7 cells. Contrastingly, phosphorylated P90RSK and JNK were increased in response to osthole in BT-474 and MCF-7 cells. 

### 3.7. Interaction between Osthole-Mediated Modulation of the PI3K/Akt and MAPK Pathways

**Figure 7:**
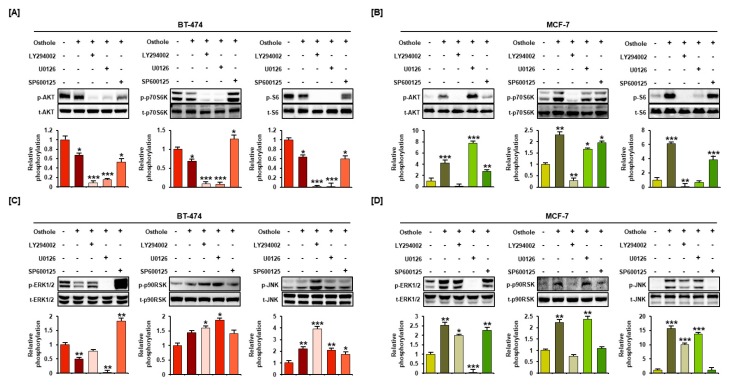
Combinational effect of pharmacological inhibitors on osthole-mediated signaling pathways in human breast cancer cells. After incubation with LY294002 (Akt inhibitor), U0126 (ERK1/2 inhibitor), and SP600125 (JNK inhibitor) for 2 h, BT-474 and MCF-7 cells were treated with 50 μM osthole for 1 h. Changes in phosphorylation of Akt, p70S6K, S6 (**A**,**B**), ERK1/2, p90RSK, and JNK (**C**,**D**) were analyzed by western blotting. The graph shows relative phosphorylation of each protein compared to that in non-treated control (0 μM). All experiments were performed in biological triplicate and the asterisks indicate statistically significant differences compared to non-treated control (* *p* < 0.05, ** *p* < 0.01 and *** *p* < 0.001).

Modulation of the PI3K/Akt and MAPK pathways by osthole was verified by pretreatment with pharmacological inhibitors including LY294002 (PI3K inhibitor, 20 μM), U0126 (ERK1/2 inhibitor, 10 μM), and SP600125 (JNK inhibitor, 20 μM). Phosphorylation of Akt, p70S6K, and S6 was completely inhibited by LY294002 and U0126 in BT-474 cells (Figure 7A). Increased Akt activation was inhibited only by LY294002. However, phosphorylation of P70S6K and S6 was blocked by LY294002 and U0126 in MCF-7 cells (Figure 7B). U0126 inhibited the phosphorylation of ERK1/2 in BT-474 and MCF-7 cells. Additionally, phosphorylation of ERK1/2 was increased by SP600125 in BT-474 cells. Moreover, phosphorylation of JNK was blocked by SP600125 and decreased by LY294002 in MCF-7 cells (Figure 7C,D).

### 3.8. Combinational Effects of Osthole with Commercial Chemotherapeutic Drugs

**Figure 8:**
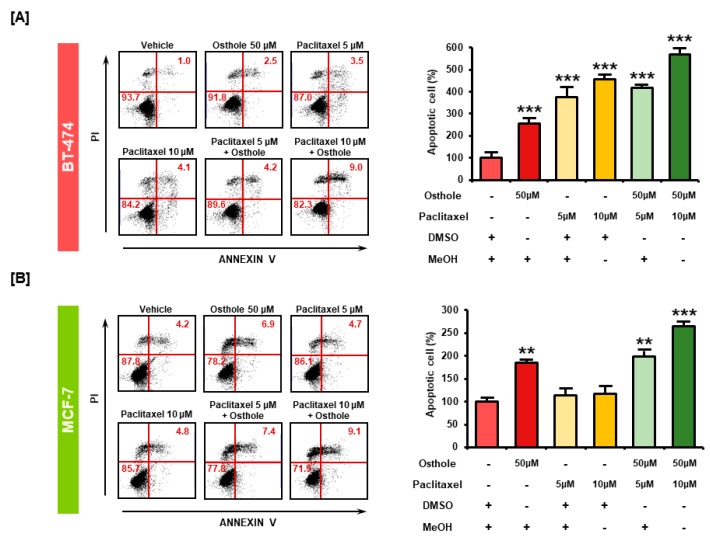
Combinational apoptotic effect of osthole and conventional chemotherapeutic agents. The proportion of apoptotic cells following treatment with osthole with and without paclitaxel was detected using Annexin V/PI staining in BT-474 (**A**) and MCF-7 (**B**). BT-474 and MCF-7 cells were incubated with osthole, paclitaxel, or a combination of both for 48 h. Data are represented in the graph as the percentage of apoptotic cells compared with vehicle-treated control (100%). All experiments were performed in biological triplicate and the asterisks indicate statistically significant differences compared to Vehicle-treated control (** *p* < 0.01 and *** *p* < 0.001).

We investigated the anti-cancer effects of combination treatment with osthole and the commercial anticancer drug, paclitaxel, in breast cancer cells using the Annexin V assay. In BT-474 cells, treatment with paclitaxel increased the number of apoptotic cells by 377% and 456% at 5 μM and 10 μM concentrations, respectively. However, combined treatment with osthole (50 μM) increased apoptosis by 418% and 569% at each concentration of paclitaxel (*p* < 0.001) (Figure 8A). In MCF-7 cells, paclitaxel increased the number of apoptotic cells by 114% and 117% at 5 μM and 10 μM concentrations, whereas combined treatment with osthole increased the apoptosis of MCF-7 cells by 199% (*p* < 0.01) and 265% (*p* < 0.001), respectively (Figure 8B). These results showed that the combinational treatment exerted higher apoptotic effect than treatment with osthole or paclitaxel alone in breast cancer cell lines.

## 4. Discussion

**Figure 9:**
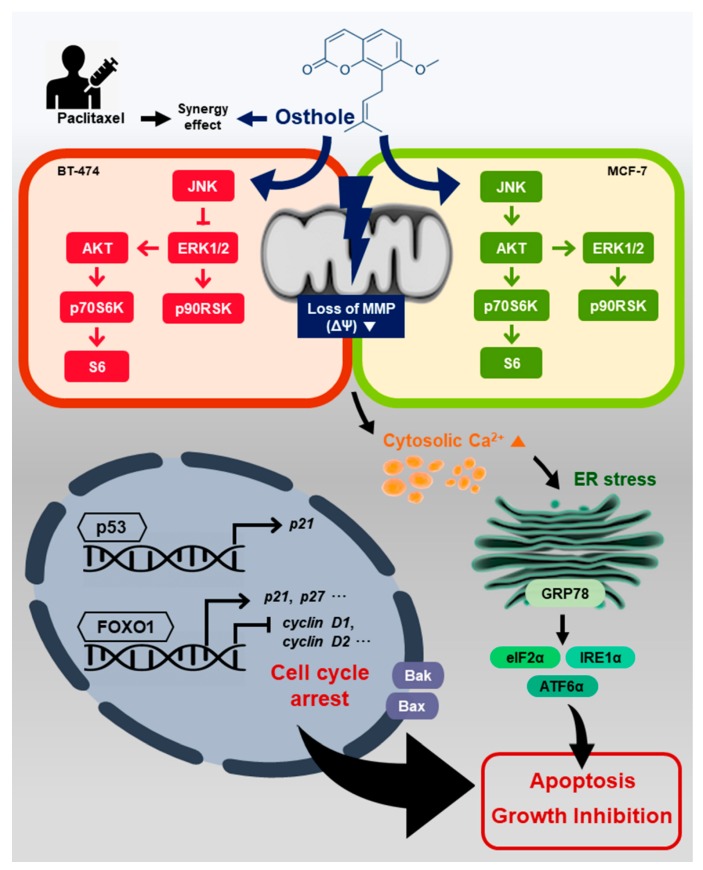
Hypothetical mechanism of action of osthole in human breast cancer cell lines. Osthole induced loss of the MMP and increased cytoplasmic calcium levels. ER stress-response proteins and pro-apoptotic proteins were increased in BT-474 and MCF-7 cells. Osthole upregulated JNK in both cell lines, downregulated the Akt and ERK1/2 signaling pathways in BT-474 cells, and upregulated these pathways in MCF-7 cells. Osthole induced growth inhibition and apoptosis in breast cancer cell lines.

We showed that osthole exerted anti-cancer effects against BT-474 and MCF-7 human breast cancer cell lines. It induced cell cycle arrest and suppressed cell proliferation. Osthole disrupted intracellular homeostasis by depolarizing the mitochondrial membrane potential and increasing cytosolic calcium levels. Moreover, osthole activated ER stress proteins and regulated the activity of the PI3K/Akt and MAPK signaling pathways, resulting in apoptosis of breast cancer cells (Figure 9).

Breast cancer, the most commonly diagnosed cancer and the second leading cause of cancer death in women, is comprised of a heterogeneous population of cells. Clinical subtypes of breast cancer are classified based on gene expression profiles [26]. Each breast cancer subtype responds differently to anti-cancer treatments [27]. Recently, molecular classification of breast cancer has been used for treatment design, resulting in improved survival rates [28]. Although, recently, the number of pathological studies of breast cancer have increased, several challenges including heterogeneity, gene mutation, and tumor microenvironment remain [29]. Particularly, tumor heterogeneity complicates the treatment of breast cancer. Therefore, it is necessary to identify a novel compound that targets multiple signaling pathways involved in breast cancer.

Natural extracts—*Ganoderma lucidum* extract, luteolin, and ginsenoside—are known to exert anti-cancer effects in human breast cancer cell lines [30,31,32]. Apigenin also inhibited cell growth and increased the number of BT-474 cells in the sub-G1 phase [33]. Studies showed that osthole inhibited cell proliferation, migration, and invasion of breast cancer cells [24]. Similarly, here, osthole inhibited cell proliferation and induced apoptosis in both breast cancer cell lines evaluated. Additionally, osthole induced sub-G1 phase arrest in BT-474 cells and G1 phase arrest in MCF-7 cells.

The cell cycle is regulated by protein complexes consisting of cyclin and cyclin-dependent kinase (CDK). In many cancers, cell cycle arrest has been considered a potential target for cancer therapies. Reduction of cyclin D inhibited tumor growth and induced cell death in esophageal, colon, and pancreatic cancer [34,35,36]. Furthermore, targeting cyclin E1 induced cell cycle arrest and apoptosis in breast cancer cells [37] and hepatocellular carcinoma cells [38]. Additionally, various anti-cancer agents exert medicinal effects by reducing the expression of CDK family proteins; e.g., flavopiridol induced cell cycle arrest through inhibition of CDKs in human breast cancer cells [39], and CYC202, a potent inhibitor of CDK2, reduced tumor growth in vitro and in vivo [40]. Likewise, here, osthole inhibited the mRNA expression of *cyclin E1*, *cyclin D1*, *CDK2*, and *CDK6* in BT-474 and MCF-7 cell lines. Furthermore, osthole suppressed the mRNA expression of *ERα*, which plays a critical role in breast cancer progression in both cell lines. ERα was shown to directly bind to TP53 and downregulate transcriptional activation of TP53 and functional genes in the p53-mediated apoptotic pathway [41,42]. In response to DNA damage, TP53 was activated and facilitated the transcription of downstream genes including *P21* [43]. P21 is a CDK inhibitor that binds to multiple CDK/cyclin complexes and PCNA [44]. The mRNA expression of *P21* is also increased by FOXOs, which are localized in the nucleus and act as transcription factors [45]. Quercetin increased FOXO1 expression, resulting in induction of P21, leading to cell cycle arrest and apoptosis in oral cancer [46]. Here, osthole upregulated the mRNA expression of *TP53*, *P21*, and *FOXO1*, resulting in cell cycle arrest and apoptosis in human breast cancer cell lines.

Mitochondria-dependent apoptosis is a major apoptotic pathway in mammalian cells. Osthole induced apoptosis via disruption of the mitochondrial membrane potential in cholangiocarcinoma [47]. Additionally, osthole induced mitochondrial permeability transition, resulting in apoptosis in human hepatocarcinoma cells [48]. Disruption of the mitochondrial membrane activated pro-apoptotic proteins—Bak and Bax—in the outer membrane, resulting in proteolipid pore formation. Cytochrome c was secreted into the cytoplasm through these pores, resulting in enhanced caspase activity [49]. Similarly to previous studies, osthole depolarized the mitochondrial membrane and increased Bax, Bak, and activity of caspase 3 and 9 in BT-474 and MCF-7 cells in our study. Thus, we concluded that osthole induces apoptosis in breast cancer cells via activation of proapoptotic factors including caspases proteins. Recent studies revealed that activation of caspase proteins could generate proteolytically-activated proapoptotic fragments [50,51,52]. Therefore, further researche on other apoptotic pathways will contribute to understanding osthole-mediated cell death mechanisms in breast cancer cells.

Calcium signaling is involved in various cellular functions including cell proliferation and apoptotic cell death [53]. Furthermore, disruption of intracellular calcium homeostasis can induce endoplasmic reticulum (ER) stress, leading to the unfolded protein response (UPR) [54]. Osthole has been shown to activate the ER stress signaling pathway in osteoblasts [55]. Similarly, we showed that the ER stress signaling proteins GRP78, IRE1α, ATF6α, and phosphorylated eIF2α were upregulated by osthole in BT-474 and MCF-7 cells. These results indicated that osthole disrupted the mitochondrial membrane potential (MMP) and calcium homeostasis, resulting in ER stress in human breast cancer cell lines.

Abnormal cell proliferation and invasion induced by alterations of signaling pathways are hallmarks of cancer [56]. The PI3K/Akt and MAPK/ERK1/2 signaling pathways are important in normal cellular physiology, and are common targets for treatment of diverse human cancers, including breast cancer [57]. Particularly, the PI3K/Akt signaling pathway plays an important role in cell proliferation, growth, and survival in cancer [58]. Therefore, the PI3K/Akt pathway is constitutively activated in several types of cancer cells; e.g., the HER2 receptor family activates the PI3K/Akt signaling pathway and leads to sustained proliferative signaling in breast cancer cells [59]. Osthole was shown to inhibit the Akt signaling pathway and induce apoptosis in HER2-overexpressing breast cancer cells [60]. Furthermore, osthole suppressed proliferation and induced apoptotic cell death via inhibition of the PI3K/Akt and ERK1/2 signaling pathways in rat glioma cells [61]. Similarly, here, osthole downregulated the phosphorylation of Akt, p70S6K, S6, and ERK1/2 in BT-474 cells.

In contrast to the results obtained using BT-474 cells, osthole activated the Akt and ERK1/2 pathways in MCF-7 cells. In contrast to the known cell proliferative properties of PI3K/Akt, Akt facilitated apoptosis rather than cell growth under pathologic conditions; e.g., rapamycin, a cytotoxic chemotherapeutic agent, induced cancer cell death via activation of Akt [62]. Reduced Akt was shown to confer resistance to oxidative stress and cellular apoptosis [63]. Similarly, the ERK signaling pathway promotes apoptosis in cell type- and stimulus-dependent manners [64]. Several phytochemicals—baicalein and kaempferol—enhanced apoptosis by activating the ERK1/2 pathway in MCF-7 cells [65]. Moreover, doxorubicin activated ERK1/2 and induced G1 phase cell cycle arrest and apoptosis in MCF-7 cells [66]. Additionally, JNK activation mediated apoptotic signaling through upregulation of pro-apoptotic genes and caspase activity [67]. In breast cancer, tocotrienol and rotenone induced apoptosis via activation of JNK [68,69]. Here, osthole increased JNK phosphorylation in both breast cancer cell lines.

The effects of combination treatment with osthole and LY294002, U0126, or SP600125 was evaluated in both breast cancer cell lines. Phosphorylation of Akt, p70S6K, and S6 was strongly suppressed by the PI3K inhibitor and by the MEK inhibitor. The ERK1/2 pathway was activated by the JNK inhibitor in BT-474. These results indicated that ERK1/2 was inhibited by JNK, and activated the Akt pathway in BT-474 cells. In MCF-7 cells, the Akt pathway was downregulated by the JNK inhibitor and ERK1/2, p90RSK, and JNK were downregulated by the PI3K inhibitor. These results suggested that osthole-induced JNK activation increased PI3K/Akt and ERK1/2 signaling, resulting in apoptosis.

The signaling discrepancies between the two breast cancer cell lines might be associated with distinctive molecular profile including receptor status [70]. Indeed, BT-474 and MCF-7 cells have been known to exert different clinical responses to the same treatment [71]. Therefore, further validation of basal activity on two major pathways and the status of receptors associated with signaling cascade should be conducted to understand the differences between the BT-474 and MCF-7 cells.

## 5. Conclusions

We demonstrated the anti-cancer effects of osthole in human breast cancer cells. Osthole inhibited cell proliferation via reduced PCNA expression and induced cell cycle arrest. It downregulated the mRNA expression of cell cycle regulators—cyclin and CDK family proteins—and upregulated the mRNA expression of *P21*, *TP53*, and *FOXO1* transcription factor. Additionally, osthole induced apoptotic cell death by disrupting the MMP and increasing intracellular calcium levels and ER stress in human breast cancer cells. Osthole also modulated the PI3K/Akt and MAPK/ERK1/2 signaling pathways. Moreover, the apoptotic cell death by paclitaxel was increased with cotreatment with osthole. Thus, osthole has great potential in human breast cancer therapy.

## Figures and Tables

**Table 1:** Primer sequence for quantitative RT-PCR.

Gene Symbol	GenBank No.	Forward Primer (5′→3′)	Reverse Primer (5′→3′)
*CCND1*	NM_053056.2	TGCTGGTTTTCTACCCAACG	AGTGCTTGGAAATGGAATGG
*CCNE1*	NM_001238.3 (Updated as NM_001238.4)	CGGCCTTGTATCATTTCTCG	TCCCCGTCTCCCTTATAACC
*CDK2*	BT006821.1	TTTGCTGAGATGGTGACTCG	AAGTAACTCCTGGCCACACC
*CDK4*	NM_000075.3 (Updated as NM_000075.4)	CCGAAGTTCTTCTGCAGTCC	CCACAGAAGAGAGGCTTTCG
*CDK6*	AK313491.1	CATTCAAAATCTGCCCAACC	TGGAAGTATGGGTGAGACAGG
*ERα*	NM_001328100.2	CAGGCTTTGTGGATTTGACC	ATTTTCCCTGGTTCCTGTCC
*P21*	NM_000389.4 (Updated as NM_000389.5)	GACTCTCAGGGTCGAAAACG	GGATTAGGGCTTCCTCTTGG
*TP53*	NM_000546.5	GTCTTTGAACCCTTGCTTGC	CCACAACAAAACACCAGTGC
*FOXO1*	NM_002015.3 (Updated as NM_002015.4)	CAGCAAGTTCATTCGTGTGC	CTGTTGTTGTCCATGGATGC
*GAPDH*	NM_001289745.3	GGCTCTCCAGAACATCATCC	TTTCTAGACGGCAGGTCAGG
